# Psychoactive and Neurobiological Effects of Ketamine in Humans: A Scoping Review of Clinical Evidence

**DOI:** 10.3390/jcm15155922

**Published:** 2026-07-29

**Authors:** Sidorela Turtulli, Eugenia Papadaki

**Affiliations:** 1 Department of Biomedical Science, School of Health and Sports Sciences, New York College, 138 Egnatia, 54622 Thessaloniki, Greece; sturtulli@nyc.gr; 2 Department of Human Nutrition and Dietetics, School of Health and Sports Sciences, New York College, 138 Egnatia, 54622 Thessaloniki, Greece

**Keywords:** treatment-resistant depression, ketamine, rapid-acting antidepressants, suicidality, synaptic plasticity, glutamatergic signaling

## Abstract

**Background/Objectives**: Ketamine has attracted interest in psychiatry due to its rapid antidepressant effects, particularly in patients with inadequate response to conventional treatments. This scoping review maps ketamine’s neuropharmacology, mechanisms of action, clinical applications, safety, and use in psychiatric disorders. **Methods**: This scoping review was conducted according to the guidelines of Preferred Reporting Items for Systematic Reviews and Meta-Analyses Extension for Scoping Reviews. PubMed, Scopus, Web of Science, and Google Scholar were searched for English-language publications addressing ketamine pharmacology, clinical applications, and psychoactive effects. Included sources comprised randomized controlled trials, observational studies, systematic reviews, and relevant mechanistic studies, while non-English publications, editorials, commentaries, and studies without sufficient methodological information were excluded. Data were extracted using a structured data-charting form and synthesized narratively and thematically. **Results**: A total of 69 studies were included in the final synthesis. Ketamine demonstrated early improvements in depressive symptoms, especially in treatment-resistant depression (TRD), although the durability of benefits varied across studies. Intravenous racemic ketamine was the most extensively investigated administration route, while intranasal esketamine represents the primary approved formulation for TRD. Mechanistically, the drug acts through N-methyl-D-aspartate receptor antagonism and downstream modulation of glutamatergic signaling, synaptic plasticity and neural connectivity. Comparisons with conventional antidepressants and neuromodulatory interventions highlighted ketamine’s faster onset of action, although variability remained regarding long-term effects and treatment strategies. **Conclusions**: Ketamine represents an important rapid-acting treatment for TRD and severe depressive episodes associated with suicidal ideation. However, information regarding long-term efficacy and safety remains limited due to study heterogeneity, short follow-up periods, and variability in dosing protocols. Further research is needed to address gaps related to long-term outcomes, maintenance strategies, and optimal dosing approaches.

## 1. Introduction

Psychoactive drugs are substances that influence mental processes such as perception, mood, cognition, consciousness, and behavior through their actions on the central nervous system [1,2]. These compounds may produce therapeutic effects in mental disorders. However, they are also associated with important health, emotional, and social concerns, including dependence, toxicity, and substance misuse. Consequently, disorders related to the use of these substances are frequently encountered in psychiatric services, emergency departments, and community healthcare settings [2].

The use of psychoactive substances dates back centuries, as humans have long consumed compounds capable of altering mood and consciousness for healing and recreational purposes. Alcohol and opiates have been recognized for their anxiolytic and sedative effects, and many major classes of psychotropic drugs employed in psychiatry today originated from early clinical observations of such effects [2,3]. During the twentieth century, advances in neuroscience and pharmacology contributed to the development of modern psychopharmacology and the increasing use of medications in mental health care. Several major discoveries emerged through examination and serendipity. One notable example was the observation by Charles Bradley in 1937 that benzedrine improved behavior and school activities in children. This finding, initially made in a medical setting, later contributed to the development of stimulant-based treatments for childhood behavioral disorders that would eventually be conceptualized under diagnoses such as hyperkinetic impulse disorder and, later, attention-deficit/hyperactivity disorder [4].

The introduction of chlorpromazine and antidepressant agents during the 1950s, including monoamine oxidase inhibitors and tricyclic molecules, marked the beginning of neuropharmacology and transformed the treatment of psychiatric disorders by introducing drug classes that were rapidly adopted into clinical practice and reshaped emotional patient care [5,6]. Subsequently, psychopharmacological research evolved toward a more detailed mechanistic understanding of drug action on defined neurotransmitter systems and receptor pathways. Psychoactive drugs are generally classified into pharmacological categories such as stimulants and depressants, based on their primary neurochemical targets and functional effects on neuronal signaling. These substances exert their actions through different neurotransmitter systems and produce distinct behavioral and physiological outcomes. For example, stimulants such as amphetamine and cocaine act through monoaminergic mechanisms involving norepinephrine, dopamine, and serotonin transporters, thereby modulating monoaminergic transmission [7]. In contrast, depressants, including benzodiazepines and barbiturates, act on the *γ*-aminobutyric acid (GABA) type A receptor–chloride ion channel complex. This enhances receptor-mediated chloride conductance, promotes membrane hyperpolarization, and reduces the probability of neuronal action potential generation [8]. Similarly, opioids produce analgesic effects through activation of opioid receptors, which are G protein-coupled receptors that induce cellular hyperpolarization [9]. On the other hand, cannabinoids act primarily through cannabinoid receptor type 1 and type 2, which are G protein-coupled receptors. Cannabinoid receptor type 1 is mainly found in the central nervous system and inhibits the release of neurotransmitters, whereas cannabinoid receptor type 2 is primarily associated with immune system cells and modulates cytokine release and immune cell migration [10]. Finally, serotonergic hallucinogens (psychedelics), such as lysergic acid diethylamide (LSD) and psilocybin, exert their effects through activation of serotonin 5-HT2A receptors, thereby modulating perception, mood, cognition, and states of consciousness [11].

Among these compounds, ketamine has gained considerable attention due to its unique neuropharmacological profile and rapid reduction in depression. Initially developed as a dissociative anesthetic, the drug is now used in anesthesiology and psychiatry [12]. Unlike conventional antidepressants such as selective serotonin reuptake inhibitors (SSRIs), ketamine acts primarily as an N-methyl-D-aspartate receptor (NMDAR) antagonist within the glutamatergic system, with effects emerging within hours rather than weeks [3,13]. It has therefore been extensively studied for treatment-resistant depression (TRD), a form of major depressive disorder (MDD) characterized by persistent depressive symptoms following inadequate response to appropriate treatment attempts with conventional therapeutic approaches, such as antidepressant medications, psychotherapy, or augmentation strategies [14]. Despite its therapeutic potential, ketamine is associated with adverse effects, including acute dissociative symptoms, perceptual disturbances such as hallucinations, and dose-dependent cognitive and psychomimetic effects. With repeated or recreational usage, it may also lead to urinary tract toxicity, including ulcerative cystitis, as well as psychological dependence. Due to its mental properties and abuse potential, ketamine is classified as a controlled substance in many countries and should only be administered under medical supervision. Overall, it represents a significant development in modern psychopharmacology and remains an active area of investigation in contemporary psychiatric research [12,13,15,16].

This scoping review aims to characterize the neuropharmacological mechanisms, psychoactive effects, clinical applications, therapeutic outcomes, and safety considerations of ketamine use in human psychiatric research. Specifically, it seeks to summarize current evidence regarding ketamine’s mechanisms of action, efficacy in psychiatric disorders, and associated risks and limitations. By integrating evidence from human clinical studies and peer-reviewed scientific literature, it provides a comprehensive overview of ketamine within modern psychopharmacology. The review follows the Preferred Reporting Items for Systematic Reviews and Meta-Analyses Extension for Scoping Reviews (PRISMA-ScR) framework and systematically maps the available evidence on ketamine’s neuropsychoactive and treatment-related effects in human research.

## 2. Methods

### 2.1. Search Strategy

A comprehensive scoping review was conducted using PubMed, Scopus, Web of Science, and Google Scholar, with searches performed from inception to 19 July 2026. Only English-language publications were considered for inclusion. In addition, relevant governmental, regulatory, and organizational sources accessed online, including the World Health Organization, European Medicines Agency, United States Drug Enforcement Administration, Health Canada, and European Union Drugs Agency, were consulted to provide contextual information regarding ketamine regulation and public health considerations. All databases were last searched, and all governmental, regulatory, and organizational online sources were last consulted on 19 July 2026. Reference lists of included studies and relevant reviews were also screened to identify additional eligible sources. The review was conducted in accordance with the Preferred Reporting Items for Systematic Reviews and Meta-Analyses Extension for Scoping Reviews (PRISMA-ScR) guidelines [17] and the study selection process was illustrated using the PRISMA 2020 flow diagram [18]. The review was not registered, and no protocol was made publicly available.

All screening and study selection were performed independently by the two authors using predefined eligibility criteria. Retrieved records were screened in multiple stages, including title, abstract, and full-text assessment. Disagreements regarding study eligibility were resolved through joint review of the full text and reassessment against the predefined inclusion and exclusion criteria. Consensus was reached after considering each study’s methodological relevance and contribution to the objectives of the review. No automation tools were used for record screening, study selection, or eligibility assessment.

The search strategy combined database-specific controlled vocabulary (e.g., MeSH terms) and free-text terms related to ketamine, its clinical applications, pharmacology, neurobiology, psychiatric indications, and safety. Boolean operators were adapted for each database. Full search strategies, including terms, operators, filters, and search dates, are provided in Supplementary Table S1.

### 2.2. Inclusion Criteria

This scoping review followed the PCC framework (Population, Concept, Context) to define eligibility criteria for included studies. Studies were selected based on the following:

- Population: Human participants from clinical or observational studies evaluating ketamine exposure or treatment outcomes.
- Concept: Ketamine as a psychoactive substance, including its pharmacological properties, mechanism of action, and central nervous system effects.
- Context: Clinical and psychiatric applications, particularly anesthesia, analgesia, and psychiatric disorders (such as major depressive disorder, treatment-resistant depression, anxiety, and post-traumatic stress disorder).
- Study types: Randomized controlled trials, observational studies, and systematic reviews. Preclinical studies were included only when they provided relevant mechanistic or translational insights.
- Language and publication criteria: Peer-reviewed publications published in English and available up to 2026 were included.

These eligibility criteria were selected to capture the breadth of human clinical evidence and relevant translational findings concerning ketamine’s pharmacological mechanisms, therapeutic applications, and safety profile.

### 2.3. Exclusion Criteria

Studies were excluded based on the following criteria:

- Population: Studies not involving human participants and lacking translational relevance to clinical outcomes.
- Concept: Studies focusing exclusively on other psychoactive substances without relevance to ketamine pharmacology or mechanism of action.
- Context: Studies not addressing clinical, psychiatric, anesthetic, or analgesic applications of ketamine, including purely experimental studies without translational relevance.
- Study types: Editorials, opinion pieces, commentaries, letters to the editor, and conference abstracts without full-text availability.
- Data availability: Studies lacking sufficient methodological detail or extractable data for synthesis.
- Language and publication criteria: Non-peer-reviewed publications and non-English-language studies were excluded.

### 2.4. Data Extraction and Thematic Analysis

Data were systematically extracted using a structured data-charting form developed a priori and piloted on a subset of studies to ensure consistency and completeness. The extraction process was performed independently by the two authors. No additional information was requested from study authors, and no automation tools were used.

The following variables were extracted from each study and synthesized using a thematic analysis approach:

- Study characteristics: Authors, year, country, study design, sample size, population.
- Ketamine-related variables: Dosage, route of administration, frequency, and duration of treatment/exposure.
- Clinical and psychiatric outcomes: Primary and secondary outcomes related to anesthesia, analgesia, or psychiatric disorders (e.g., depression, treatment-resistant depression, anxiety, post-traumatic stress disorder).
- Mechanistic and neurobiological findings: Effects on neurotransmitter systems, synaptic plasticity, NMDAR antagonism, glutamatergic signaling, and other relevant central nervous system mechanisms.
- Methodological details: Inclusion/exclusion criteria, study setting, follow-up duration, and reported study limitations.

All reported outcomes related to ketamine’s neuropharmacological effects, psychoactive properties, clinical efficacy, and safety were extracted where relevant to the review objectives. When information was unclear or unavailable, it was recorded as not reported in the original publication. Extracted data were reviewed and organized according to study characteristics, ketamine-related variables, outcomes, and methodological features before synthesis.

Extracted data were synthesized using a thematic analysis approach, grouping studies according to key themes: pharmacological mechanisms, clinical applications, psychiatric outcomes, and neurobiological effects. Studies were allocated to these synthesis categories based on their research objectives, study characteristics, ketamine-related variables, and reported clinical or neurobiological outcomes. Narrative summaries were generated for each theme, and Tables were used to present study characteristics and key findings for clarity. Visual mapping was employed where appropriate to depict relationships between ketamine mechanisms and clinical outcomes. A narrative and thematic synthesis approach was selected because of the diversity of study designs, populations, interventions, and outcome measures, which precluded quantitative pooling of results. In accordance with scoping review methodology, critical appraisal was not performed, as this review aimed to map the breadth and characteristics of evidence rather than evaluate methodological quality or estimate treatment effects.

## 3. Results

### 3.1. Study Selection and Characteristics

**Figure 1:**
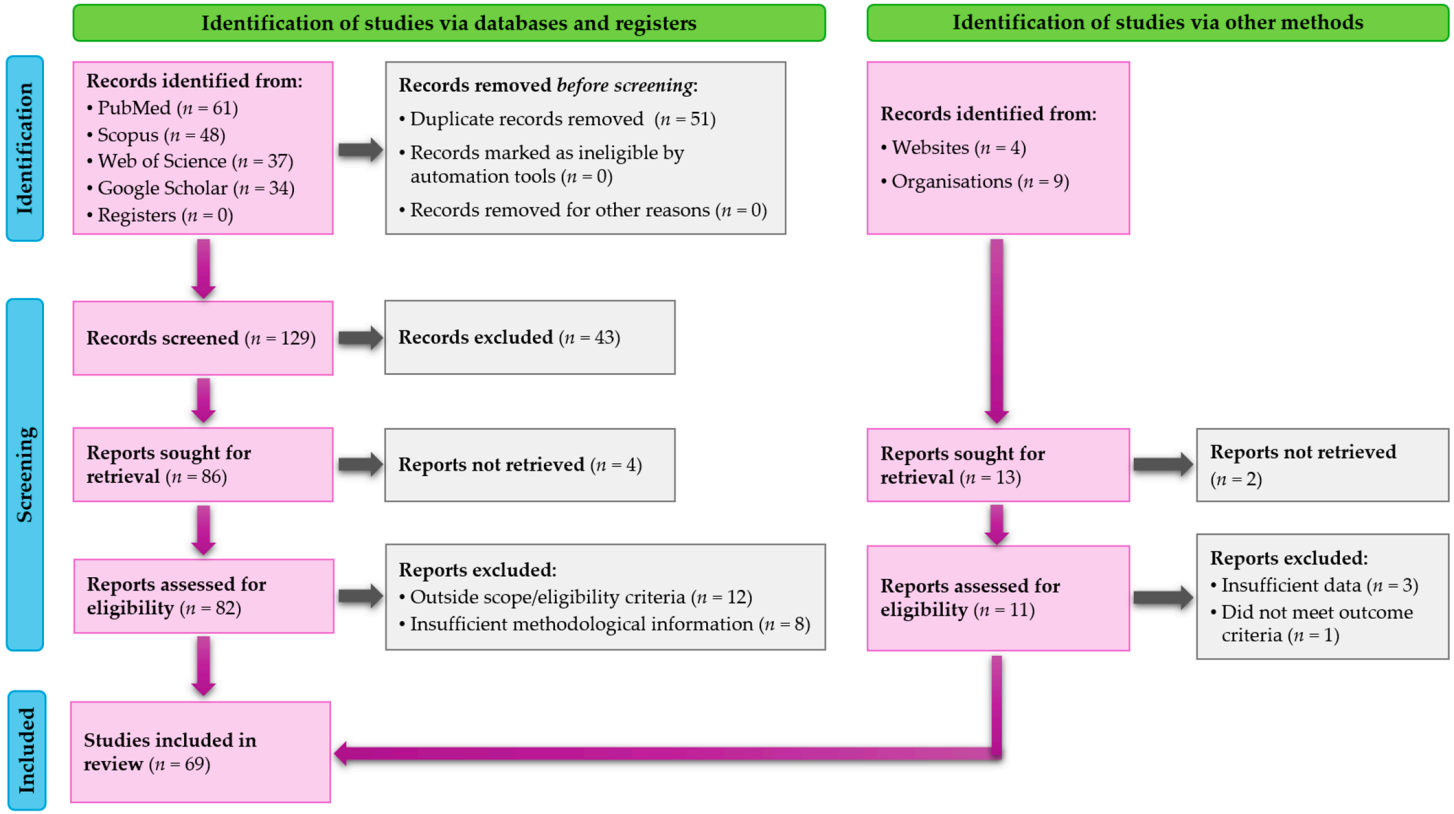
PRISMA 2020 flow diagram illustrating the study selection process, adapted from the original template [18] under the CC BY 4.0 license. The review was conducted in accordance with the PRISMA-ScR reporting guidelines [17].

Figure 1 illustrates the study selection process using the PRISMA 2020 flow diagram. A total of 180 records were identified through database searches: 61 from PubMed, 48 from Scopus, 37 from Web of Science, and 34 from Google Scholar. Additionally, 13 records were identified through other sources, including websites (*n* = 4) and organizational sources (*n* = 9). After removing duplicates (*n* = 51), 129 records remained for screening based on titles and abstracts. Of these, 43 records were excluded due to irrelevance or failure to meet eligibility criteria, leaving 86 reports for full-text assessment. During full-text retrieval, 4 database records and 2 records from other sources could not be retrieved.

Full-text assessment resulted in the exclusion of additional reports. Specifically, 12 reports were excluded because they did not meet the scope/eligibility criteria, 8 were excluded as they lacked sufficient methodological information required for data extraction, 3 contained insufficient data for extraction, and 1 did not meet specific outcome criteria. Ultimately, 69 studies met the inclusion criteria and were included in the scoping review. These studies were subsequently synthesized thematically.

The evidence base comprised heterogeneous study designs, reflecting the broad scope of ketamine research across clinical and experimental domains. Included study types consisted of randomized controlled trials, observational studies, narrative reviews, systematic reviews, and mechanistic studies providing translational insights. Most studies focused on ketamine’s applications in anesthesia, analgesia, and psychiatric indications, particularly MDD and TRD. Considerable methodological variability was observed across studies, including differences in design, sample sizes, populations, dosing regimens, routes of administration, and outcome measures. While the majority of findings derived from adult human populations, a smaller proportion of studies contributed translational or mechanistic insights supporting neurobiological interpretation. Review articles provided broader synthesis of existing evidence, whereas primary studies contributed empirical data on clinical outcomes, safety, and mechanistic insights.

### 3.2. Routes of Ketamine Administration

**Figure 2:**
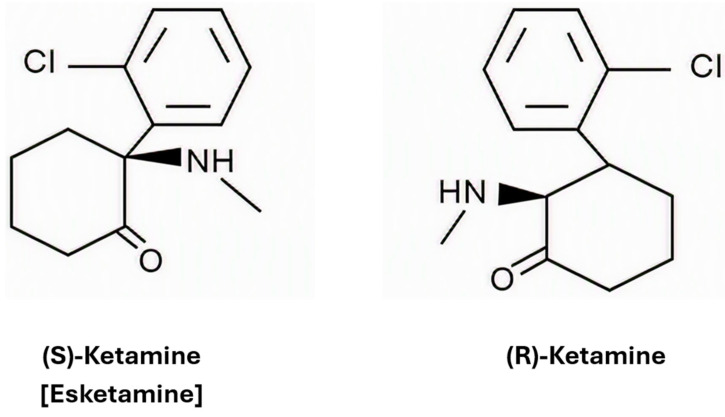
Chemical structure of ketamine enantiomers [20].

Ketamine is a phencyclidine derivative originally designated “CI-581,” with the molecular formula C_13_H_16_ClNO and a molecular weight of approximately 238 Da. It is considered a weakly basic compound with pKa ≈ 7.5. Clinically, ketamine is administered as a racemic mixture consisting of equal proportions of the (S)- and (R)-enantiomers (Figure 2). These enantiomers have identical molecular connectivity but differ in their three-dimensional spatial configurations [19,20]. The (S)-enantiomer, known as esketamine, is available as a separate medicinal product and is primarily used for TRD, owing to its enhanced potency and distinct pharmacological profile compared to the (R)-enantiomer [20,21].

Racemic ketamine is available as ketamine hydrochloride, the salt form that improves solubility and activity [22]. In the United States, the injectable formulation is commonly marketed as Ketalar^®^ [23], while European regulatory records list nationally authorized racemic ketamine products under the same brand name in several countries [24]. Other marketed formulations of racemic ketamine include Ketanest^®^, Ketaset^®^, Ketaject^®^, Ketofen^®^, Ketamine 500^®^, and Imalgen^®^ [22]. In contrast, the S-enantiomer, esketamine, is marketed in Europe under names such as Ketanest-S^®^ and S-ketamin “Pfizer”^®^ [25].

**Figure 3:**
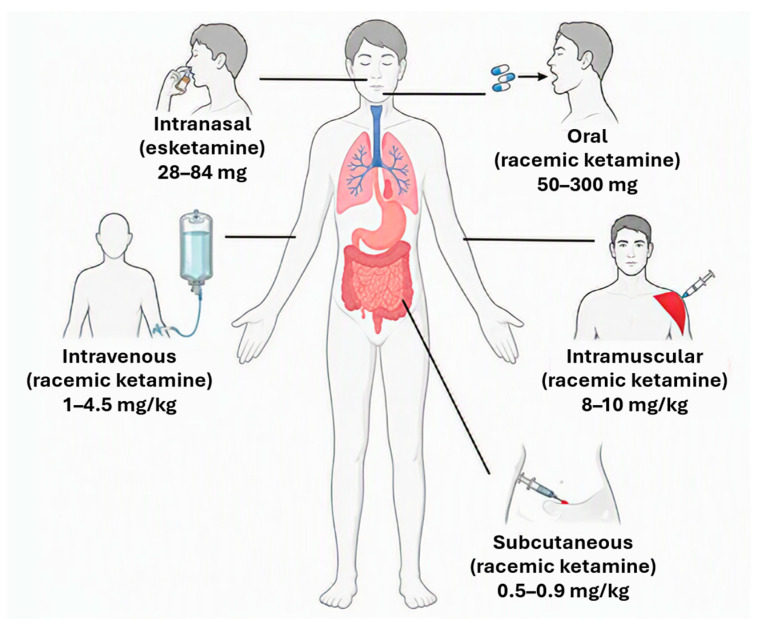
Main routes of administration of racemic ketamine and esketamine.

As illustrated in Figure 3, racemic ketamine may be administered via multiple routes, including intravenous, intramuscular, oral, and subcutaneous. These routes differ in bioavailability and onset of action, influencing ketamine application across anesthesia, analgesia, and psychiatric treatment settings [26]. Conversely, although esketamine is the only formulation approved for intranasal use against TRD, the administration of racemic ketamine via this route remains largely off-label [27,28].

**Figure 4:**
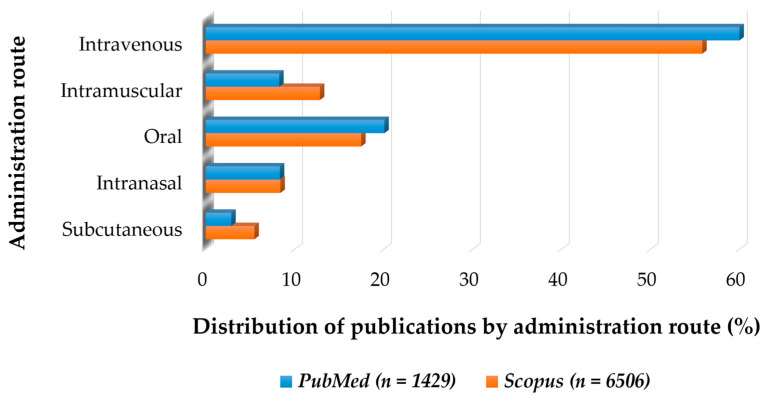
Distribution of primary research publications on racemic ketamine and esketamine by administration route in PubMed (*n* = 1429) and Scopus (*n* = 6506). Percentages show the proportion of studies per database. Studies testing multiple routes are counted in each category. Blue bars represent PubMed and orange bars represent Scopus.

The distribution of primary research publications on the administration routes of racemic ketamine and esketamine in PubMed (*n* = 1429) and Scopus (*n* = 6506) revealed notable differences, reflecting the broader literature from which the studies screened for the present review were selected. As shown in Figure 4, intravenous administration is the most extensively investigated route in both databases, accounting for approximately 60% of publications in PubMed and 56% in Scopus. Oral administration remains the second most common, representing about 20% in PubMed and 17% in Scopus. The intramuscular, intranasal, and subcutaneous approaches have been studied less frequently, with intramuscular injections being slightly more prevalent in Scopus (13%) compared to PubMed (8%). These findings highlight the prominence of intravenous and oral routes in current research. Each of the five administration routes is subsequently examined in detail to better understand the specific applications, advantages, and limitations.

Intravenous administration of racemic ketamine is the most extensively investigated route owing to its immediate onset, complete bioavailability, and precise dose titration. Dissociative symptoms typically emerge within 10–30 s, reaching peak intensity within minutes and lasting approximately 5–15 min. In clinical practice, dosing in adults and adolescents varies between 1 mg/kg and 4.5 mg/kg, administered as a slow bolus or short infusion. This route is frequently used in emergency, surgical, and psychiatric settings. However, it requires strict medical supervision due to dissociation, transient perceptual disturbances, and potential hemodynamic effects such as increases in blood pressure and heart rate [26,29].

When intravenous access is impractical, intramuscular delivery of racemic ketamine provides rapid systemic absorption and pharmacodynamic effects. Specifically, peak plasma concentrations can be reached within 5–30 min with a bioavailability of approximately 93%. Typical dosing is approximately 8–10 mg/kg. Variability in absorption can occur depending on muscle perfusion and individual patient factors. On the other hand, local injection site reactions, such as pain or bruising, may also arise [26,29].

For outpatient or longer-term treatment strategies, oral racemic ketamine intake is a non-invasive route. It undergoes extensive first-pass metabolism, resulting in reduced systemic exposure and norketamine as a major circulating metabolite [30,31]. Randomized controlled trials have used repeated dosing at approximately 1 mg/kg, with meta-analytic evidence indicating antidepressant efficacy [31]. In TRD, case series highlight flexible dosing from 50 mg every 3 days up to 300 mg, with modest response rates and a substantial proportion of non-responders. Oral racemic ketamine is generally well tolerated, with dissociation, dizziness, and blurred vision among the most commonly reported effects, and no reported serious safety concerns, abuse, or dependence [32].

Intranasal delivery has emerged as a relevant route in psychiatric therapy, especially for TRD. This is due to its ability to enable prompt central nervous system exposure while avoiding first-pass metabolism and facilitating brain targeting of antidepressant agents such as ketamine and its enantiomers. The nasal cavities provide direct and indirect pathways to the brain via the olfactory and trigeminal nerves, allowing more efficient central delivery than conventional systemic administration [33]. Intranasal racemic ketamine has been investigated in patients with MDD using a 50 mg ketamine hydrochloride formulation, demonstrating rapid activity and good tolerability [34]. Even so, intranasal esketamine is the formulation that has been most extensively evaluated in large randomized clinical trials and has consequently been approved by the United States Food and Drug Administration (FDA) as well as the European Medicines Agency for the handling of TRD [14,28].

It has been found that intranasal esketamine acts swiftly in patients with TRD, with significant symptom improvement reported within the first week of treatment at doses of 28–84 mg administered twice weekly. The 56- and 84-mg doses achieve plasma esketamine concentrations comparable to those observed after intravenous esketamine administration and within the pharmacokinetic range associated with intravenous racemic ketamine dosing [21]. More recently, a systematic review and meta-analysis reported that both intravenous racemic ketamine and intranasal esketamine significantly improve depressive symptoms, although intravenous racemic ketamine may act faster [27].

Subcutaneous administration has been investigated in depression, including TRD, using both racemic ketamine and esketamine, although most available scientific evidence is based on racemic ketamine. Fast antidepressant effects have been investigated following single and repeated dosing, with response and remission rates ranging from approximately 50% to 100% and predominantly transient dissociative and cardiovascular adverse effects [35]. In clinical trials, racemic ketamine has also been given via multiple injections over several weeks with flexible dosing in the range of 0.5–0.9 mg/kg, demonstrating the feasibility of protocol-driven administration [36]. Consequently, subcutaneous delivery has been explored as a practical alternative to the intravenous route [35,36].

Safety considerations are essential in guiding ketamine use and patient selection. It is not recommended during pregnancy due to insufficient safety data and potential fetal risk. During labor or delivery, its use is generally reserved for emergency situations when alternative options are not feasible. In breastfeeding, ketamine appears in low concentrations in milk, with limited expected infant exposure, although consumption with caution is still advised. Beyond these specific populations, it has wide applications across anesthesia, procedural sedation, pain management, and critical care, but requires monitoring of cardiovascular, respiratory, and neuropsychiatric status due to potential adverse effects. Administration should occur in controlled clinical settings with individualized dosing to ensure safe and effective function [37].

### 3.3. Pharmacodynamics, Pharmacokinetics, and Metabolism of Ketamine

**Figure 5:**
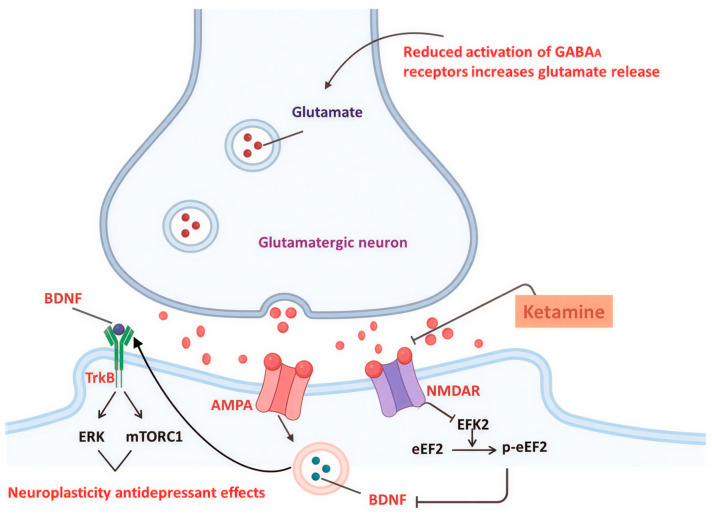
Ketamine’s mechanism of action at the glutamatergic synapse, as proposed in the recent literature [14,39]. AMPA: *α*-amino-3-hydroxy-5-methyl-4-isoxazolepropionic acid; BDNF: brain-derived neurotrophic factor; eEF2: eukaryotic elongation factor 2; ERK: extracellular signal-regulated kinase; GABA_A_: *γ*-aminobutyric acid type A; mTORC1: mechanistic target of rapamycin complex 1; NMDAR: N-methyl-D-aspartate receptor; TrkB: tropomyosin receptor kinase B.

This active ingredient primarily acts as a non-competitive and use-dependent antagonist of NMDARs by binding to the intrachannel phencyclidine site. By reducing NMDAR activity and calcium influx, ketamine produces dissociative anesthetic and analgesic effects [38]. Beyond classical intrachannel blockade, the drug may also exhibit subunit-dependent effects, with differential sensitivity among GluN2A/B- and GluN2C/D-containing receptors, and can induce allosteric inhibition through stabilization of receptor conformations that reduce activation independently of pore blockade [29]. A schematic representation of these mechanisms, including brain-derived neurotrophic factor (BDNF)–tropomyosin receptor kinase B (TrkB) and mechanistic target of rapamycin (mTOR)-related pathways, is provided in Figure 5 [14,39].

NMDAR blockade appears to reduce activity of GABAergic interneurons, resulting in a transient increase in cortical glutamatergic signaling in specific regions and enhanced α-amino-3-hydroxy-5-methyl-4-isoxazolepropionic acid (AMPA) receptor activation [14,38,39]. This is associated with strengthened synaptic plasticity, dendritic spine remodeling, and BDNF expression, and is thought to underlie the rapid antidepressant response [14]. BDNF binds to its high-affinity receptor TrkB and activates multiple intracellular signaling cascades, including the extracellular signal-regulated kinase (ERK) pathway and mechanistic target of rapamycin complex 1 (mTORC1). These pathways regulate activity-dependent protein synthesis, with mTORC1 serving as a key mediator of ketamine- induced synaptogenesis and structural plasticity in the prefrontal cortex [39]. In parallel, NMDAR inhibition reduces phosphorylation of eukaryotic elongation factor 2 (eEF2) via decreased eEF2 kinase activity. This relieves translational inhibition and increases BDNF production, thereby supporting neuroplasticity. Notably, AMPA receptor–dependent plasticity converges on intracellular signaling pathways involving TrkB, ERK, mTORC1, and eEF2, which drive synaptic remodeling and antidepressant mechanisms [14,29,39].

Although glutamatergic signaling remains the principal framework for explaining ketamine’s properties, recent preclinical evidence suggests that additional mechanisms may contribute. In particular, adenosine signaling has emerged as a potential mediator of ketamine’s antidepressant action. Experimental studies have shown that ketamine induces swift increases in extracellular adenosine within mood-regulating brain regions, including the medial prefrontal cortex and hippocampus, with activation of adenosine A1 and A2A receptors appearing necessary for its properties [40]. These discoveries hint that adenosine signaling may act alongside glutamatergic pathways to promote synaptic plasticity and emotional responses, although further studies are required to establish its medical application.

In addition to its pharmacodynamic effects, ketamine’s physicochemical properties facilitate extensive distribution. The substance is highly lipophilic (approximately five times more than thiopental) and exhibits low plasma protein binding (10–30%). Pharmacokinetics are commonly described using a three-compartment model, with an estimated central compartment volume of approximately 70 L and a steady-state volume of distribution of about 200 L (≈2.3 L/kg). Systemic clearance is high (1000–1600 mL/min or 12–20 mL/min/kg), consistent with liver blood flow dependence, and the terminal half-life is approximately 2–3 h. Elimination rates may be modestly higher in women than in men in some studies [38].

In terms of metabolism, the compound undergoes extensive biotransformation, predominantly in the liver. Cytochrome P450 isoenzymes, especially CYP3A4 and CYP2B6, catalyze the demethylation of ketamine to form norketamine, its principal metabolite. Subsequent reactions yield dehydronorketamine and hydroxyketamine derivatives, with additional hydroxylation mediated by CYP2A6. These metabolites are ultimately conjugated and eliminated via urinary and biliary excretion. Although the liver is the primary site of metabolism, metabolic activity has also been reported in the kidneys, intestines, and lungs. Following intravenous administration, norketamine appears in plasma within 20–30 min, peaks at approximately 30 min, and remains detectable for several hours. Continuous infusion may lead to metabolite accumulation, prolonging pharmacological effects and potentially reducing the need for repeated dosing [29]. Beyond its metabolic profile, ketamine also interacts with opioid, monoaminergic, and cholinergic systems [39]. Furthermore, it modulates descending pain inhibitory pathways involving serotonergic and noradrenergic projections from the rostral ventromedial medulla and locus coeruleus, contributing to analgesic effects through enhanced endogenous pain control [41].

### 3.4. Human Clinical Trials of Ketamine

Ketamine has gained substantial clinical interest in psychiatry due to its fast-acting properties against mood regulation with considerable improvement occurring within hours in some patients. This distinguishes ketamine from conventional antidepressants, which act through monoaminergic modulation (serotonin, norepinephrine, and dopamine) [42]. The early response is particularly relevant in severe depressive states, including acute suicidal ideation, where timely symptom reduction may be critical for stabilization [14].

One of the most extensively studied indications for ketamine is TRD, a subtype of MDD characterized by inadequate response to two or more antidepressant treatments administered at adequate doses and durations [13]. Across randomized controlled trials, the drug has consistently demonstrated short-term benefits [13,14]. Reductions in depressive symptom severity have been reported across various study designs, although sustained remission is less consistent, with effects often decreasing over time. Beyond TRD, ketamine has also been explored for post-traumatic stress disorder and anxiety symptoms, though the strength of evidence varies [43,44].

The drug has also been evaluated in combination with psychotherapy, mainly in TRD, where it may enhance and prolong the healing properties. To contextualize the current scientific base, Table 1 summarizes key clinical trials, dose-ranging analyses, and systematic reviews evaluating ketamine and esketamine across MDD, TRD, and related psychiatric and pain-related conditions.

Across administration routes, this agent appears to produce a consistent pattern of early symptom improvement. Intravenous ketamine has been associated with a rapid reduction in suicidal ideation, although these effects diminish over time, with only modest benefit persisting at longer follow-up compared with midazolam [45]. Similarly, intranasal delivery exhibits a comparable early response profile, showing superiority over placebo following single dose. However, these observations are transient and typically attenuate over subsequent days [34]. Consequently, the findings suggest a robust yet temporally constrained therapeutic signal across delivery modalities.

When treatment exposure is extended beyond a single administration, early action may be reinforced, although durability remains inconsistent. Repeated intravenous infusion protocols yield greater initial symptom reduction relative to control conditions. Nevertheless, the difference between groups decreases over time, suggesting that the initial response is not maintained [46]. A similar finding was reported in short-course repeated regimens, which show within-group improvement but no significant superiority over active comparators such as midazolam [47]. The observed profile raises the possibility that non-specific factors, including expectancy, sedation, and anxiety reduction, may contribute meaningfully to the medical inferences, while comparator selection can substantially influence response estimation in randomized psychiatric trials.

Open-label investigations extend these findings to clinically complex populations, including individuals with advanced cancer and comorbid depressive symptoms. Symptom improvement is typically observed during the acute treatment phase [48]. Yet, the lack of control conditions limits causal interpretation and prevents firm attribution of these changes specifically to ketamine.

From a formulation perspective, treatment structure appears a key determinant of treatment durability. Intranasal esketamine, administered at 28 mg, 56 mg, and 84 mg within repeated dosing and maintenance protocols, has been shown to produce dose-dependent effects. Under controlled conditions, therapeutic influence may persist for up to approximately nine weeks, a duration that contrasts with the more transient impacts typically observed following single-administration ketamine protocols [21].

Results from meta-analyses propose a consistent antidepressant signal across studies. Peak effects are usually observed 24 h after drug intake, with measurable benefits persisting for up to approximately one week [49]. Although repeated dosing may increase overall response magnitude, definitive conclusions regarding optimal long-term strategies are limited by variability in study design, dosing schedules, and populations. Intravenous ketamine at approximately 0.5 mg/kg remains the most consistently effective regimen for depressive symptoms in TRD populations. In contrast, lower slow-infusion strategies (0.1–0.3 mg/kg) are more commonly used in analgesic settings and appear to be associated with improved tolerability [26].

The studies summarized in Table 1 indicate that ketamine is frequently associated with rapid antidepressant effects, with apparent improvement often reported within 24 h of administration. However, the available evidence is derived predominantly from short-term randomized trials, with comparatively fewer studies evaluating long-term outcomes. Common methodological limitations include modest sample sizes, brief follow-up periods, and highly selected patient populations, all of which may restrict external validity. Additional heterogeneity in dosing regimens, routes of administration, outcome measures, and comparator conditions further limit direct cross-study comparisons. Furthermore, the use of active psychoactive comparators in several trials may reduce between-group differences and attenuate observed response sizes despite within-group improvements. Overall, although the available literature consistently supports prompt mood improvement after ketamine delivery, uncertainties remain regarding the durability of effect, optimal maintenance strategies, and comparative effectiveness across different treatment approaches.

### 3.5. Ketamine Compared with Established Treatments for Major Depressive Disorder and Treatment-Resistant Depression

**Figure 6:**
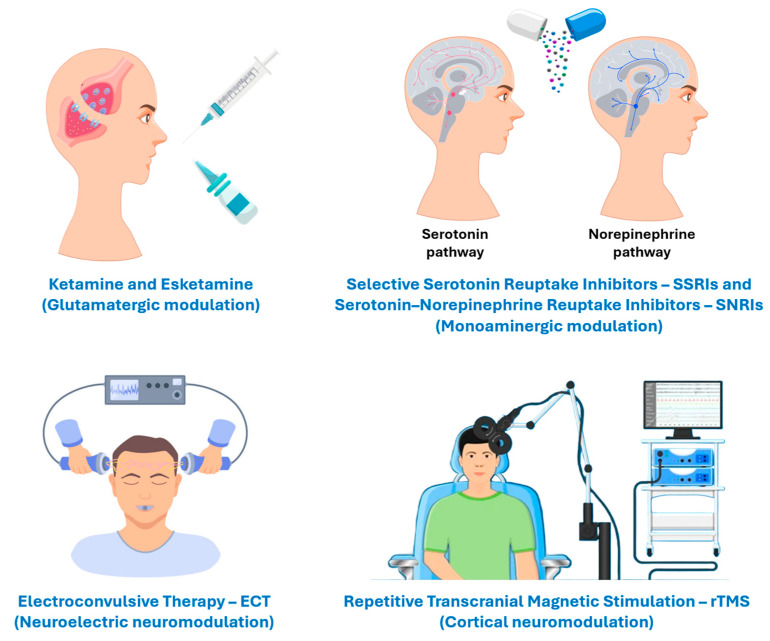
Overview of established treatment approaches for depression.

To place ketamine within the context of current treatments for depression, comparisons with established pharmacological and neuromodulatory interventions are presented. Figure 6 provides a conceptual overview of the main therapies against depression, illustrating racemic ketamine, esketamine, monoaminergic antidepressants, and non-pharmacological interventions within a simplified clinical framework.

Table 2 summarizes the principal clinical characteristics of these interventions, including onset of action, efficacy in TRD, effects on suicidal ideation, duration of response, and mechanisms of action.

Current evidence suggests broadly similar short-term response and remission rates between intravenous racemic ketamine and intranasal esketamine. Nonetheless, available studies are predominantly observational, and direct randomized comparisons remain limited. A recent systematic review and meta-analysis reported no statistically significant differences in response or remission rates between the two treatments. Nonetheless, several included studies have shown that intravenous ketamine may produce antidepressant effects more swiftly than intranasal esketamine. Interpretation of these findings should be made cautiously because the available evidence is derived predominantly from observational studies and direct randomized comparisons remain limited [27].

Importantly, the comparisons presented in Table 2 should not be interpreted as indicating superiority of one intervention over another but rather as summarizing evidence derived from different clinical contexts, study populations, and therapeutic objectives. Conventional antidepressants, particularly selective serotonin reuptake inhibitors (SSRIs) and serotonin–norepinephrine reuptake inhibitors (SNRIs), remain first-line pharmacological treatments for MDD. TRD, generally defined as persistent symptoms despite adequate antidepressant trials, represents a clinically distinct population requiring alternative or adjunctive strategies [50]. Ketamine and esketamine have been investigated predominantly in TRD rather than in broader MDD populations [27]. Therefore, differences in efficacy, onset of action, and durability of clinical benefit across treatment modalities should be interpreted in light of differences in study populations, clinical indications, therapeutic objectives, and maintenance strategies, rather than as direct evidence of therapeutic superiority.

In contrast to conventional monoaminergic psychoactives, such as SSRIs and SNRIs, glutamatergic therapies like ketamine are distinguished by their rapid onset of action [57,58]. Although SSRIs and SNRIs remain among the most commonly prescribed pharmacological remedies for MDD [59], they typically require 2 to 6 weeks to manifest a positive influence, which presents significant limitations, especially in TRD cases. For example, fluoxetine, a widely used SSRI, showed no significant effects after a single dose or even after 7 days of repeated administration, with depression reduction only appearing after 14 days of continuous treatment [58]. Additionally, SNRIs act through the inhibition of both serotonin and norepinephrine reuptake [59], which highlights their role as a psychoactive option notwithstanding their delayed onset.

Ketamine-based treatments can also be integrated with conventional antidepressant strategies in TRD. For example, esketamine combined with an SNRI was associated with lower rates of mortality, hospitalization, and depressive relapse than esketamine combined with an SSRI. However, the SSRI combination was associated with slightly fewer suicide attempts [57]. These observations point out the complementary roles of SSRIs, SNRIs, and ketamine-based interventions in the management of depressive disorders.

Beyond the established therapies, emerging psychedelic-assisted interventions have also attracted considerable interest in handling psychiatric disorders. Psilocybin, a serotonergic psychedelic, exerts its primary activity through agonism at the 5-hydroxytryptamine receptor 2A receptor, enhancing cortical connectivity and emotional processing. Clinically, psilocybin has demonstrated significant efficacy in reducing depressive symptoms, particularly in patients with TRD and end-of-life anxiety. Similarly, LSD shares core serotonergic mechanisms with psilocybin but differs in receptor specificity and subjective effects, and has shown promise in reducing anxiety, depression, and addictive behaviors. In contrast, 3,4-methylenedioxymethamphetamine primarily acts by promoting monoamine release and enhancing emotional engagement, making it suitable for trauma-focused psychotherapy in post-traumatic stress disorder [60]. Despite encouraging data, these interventions remain evolving approaches whose optimal roles continue to be defined.

Among established non-pharmacological interventions, electroconvulsive therapy (ECT) and repetitive transcranial magnetic stimulation (rTMS) represent important treatment options for severe depression and TRD, although their clinical indications, evidence bases, and treatment characteristics differ [53,54,56]. Recent preclinical studies have suggested that ketamine and ECT may share convergent mechanisms involving adenosine signaling, complementing previously described glutamatergic pathways [40]. However, mechanistic similarities do not necessarily translate into equivalent clinical profiles.

Randomized clinical trials directly comparing ketamine with ECT have demonstrated that both interventions can produce substantial reductions in depressive symptoms and suicidal ideation in patients with severe depressive illness. In a recent randomized controlled trial, ketamine and ECT both produced significant clinical improvement. Ketamine was associated with a more rapid antidepressant and anti-suicidal response, whereas ECT achieved comparable improvement across the acute treatment course [53]. Although some patients maintain improvement after an acute ECT course, relapse following completion of an acute ECT course remains common, particularly in the absence of continuation treatment. Continuation or maintenance ECT may prolong remission in selected patients but requires repeated administration and represents a relapse-prevention strategy rather than evidence that the initial acute ECT effect is inherently long-lasting [54]. The adverse-effect profiles also differ, with ECT being associated with transient cognitive adverse effects, whereas ketamine is more commonly associated with short-term dissociative symptoms and other transient adverse events [53].

Compared with ECT and ketamine-based interventions, rTMS represents a non-invasive neuromodulation approach that is primarily used in TRD and selected MDD populations. A retrospective naturalistic study comparing ketamine and rTMS in patients with TRD demonstrated significant symptom improvement following both interventions, with no significant differences in overall outcomes between groups [61]. Beyond antidepressant effects, rTMS has also demonstrated potential benefits for suicidal ideation. A meta-analysis of randomized controlled trials found that rTMS interventions were associated with significant reductions in suicidal ideation compared with control conditions, although heterogeneity in stimulation parameters, treatment protocols, and patient populations limits definitive conclusions [55].

The durability of treatment effects across ketamine, ECT, and rTMS should be interpreted cautiously. Long-term outcomes depend not only on the acute intervention itself but also on treatment scheduling, maintenance strategies, illness characteristics, and the definition of clinical outcome. Intermittent rTMS sessions administered after an acute treatment course may help maintain clinical response in some patients. However, optimal schedules, duration of treatment, and patient selection criteria remain uncertain and require further controlled investigation [56].

Regardless of its favorable profile to express symptom reduction, ketamine remains associated with several limitations. Although improvement may occur after a single administration, repeated dosing is often required to sustain clinical benefit, while long-term comparative evidence remains limited [27]. Similarly, repeated administration or maintenance approaches are commonly required for ketamine-based treatments, whereas continuation or maintenance ECT may reduce relapse risk in selected patients. Therefore, available evidence supports differences in onset of action and acute efficacy between interventions but does not establish a simple hierarchy regarding long-term durability.

## 4. Discussion

Ketamine acts promptly in depressive populations, including TRD, often within hours to 1 day in randomized controlled trials [34,46]. This quick response is especially relevant in acute depressive presentations, where urgent symptom relief is required [14]. However, sustaining this impact over time will likely require maintenance strategies, including combined pharmacological and psychotherapeutic approaches. Additionally, several studies also reported reductions in suicidal ideation, including among medically complex populations such as patients with advanced cancer [48]. Repeated administration of ketamine may help sustain the influence beyond single dosing, although relapse commonly occurs after medication discontinuation, with benefits typically diminishing over several weeks [46,49].

Another promising approach to sustaining response is ketamine-assisted psychotherapy (KAP), which combines ketamine administration with structured psychotherapeutic interventions delivered before, during, and/or after treatment. Existing data indicate that KAP may help prolong antidepressant effects, enhance patient engagement, facilitate mental processing, and potentially reduce relapse by integrating ketamine-induced healing experiences into ongoing psychotherapy. Yet, the current verdicts remain limited by small sample sizes, heterogeneous modalities and ketamine delivery protocols, and the lack of standardized treatment frameworks. Consequently, larger randomized controlled trials are needed to establish the use of KAP and determine the optimal approaches for maintaining the treatment influence [62].

As this scoping review provides a narrative synthesis of heterogeneous studies rather than a quantitative meta-analysis, direct comparisons among ketamine formulations, administration routes, and study designs should be interpreted with caution. Across randomized clinical trials, ketamine’s speed of action is apparent. Nonetheless, differences between single and repeated dosing are inconsistent at primary endpoints such as 24-h outcomes, and time-to-relapse often shows no significant between-group differences apart from early symptom improvement [46]. In addition, comparative studies with active treatments suggest that ketamine’s effects are robust but temporally limited, with no clear evidence of sustained superiority over time [49]. In naturalistic comparisons with rTMS, both interventions improve depressive symptoms, although investigations are constrained by non-randomized designs and small sample sizes [61].

Despite the promising findings on ketamine’s activity, several methodological limitations temper the strength of current evidence. Variability across randomized controlled trials includes differences in dosing regimens, administration routes, outcome measures, and follow-up periods. This heterogeneity complicates interpretation of the available findings. Additionally, many studies face challenges such as limited blinding, small sample sizes, and comparisons with different control conditions, which may introduce expectancy bias. Although early symptom improvement is consistently observed, the long-term effectiveness and optimal therapeutic strategies remain uncertain, with symptoms often returning after stopping treatment. These limitations highlight the need for standardized protocols and larger, well-controlled studies to better understand how ketamine can be successfully integrated into depression management.

Consistent with the objectives of a scoping review, the present study aimed to comprehensively map the available evidence on ketamine rather than quantitatively synthesize outcomes or formally evaluate methodological quality. This approach enabled the integration of diverse study designs and provided a broad overview of current evidence while highlighting areas requiring further research. However, because no quantitative synthesis or formal critical appraisal was undertaken, the findings should be interpreted as a descriptive overview of the available literature rather than as estimates of comparative treatment effectiveness.

At both mechanistic and clinical levels, ketamine differs from monoaminergic substances through its early engagement of glutamatergic plasticity pathways [39]. Current evidence suggests that racemic ketamine and esketamine demonstrate broadly similar antidepressant outcome. Nevertheless, limited direct head-to-head comparisons preclude firm conclusions regarding their relative roles in clinical practice [63]. Across neuromodulatory treatments, these glutamatergic interventions produce faster symptom reduction than ECT or rTMS, which require repeated sessions for full effect [53,61].

Overall, the existing data support ketamine as a useful rapid-acting intervention for TRD and other severe depressive presentations, particularly when immediate symptom relief is required. Nevertheless, uncertainties regarding the durability of response, optimal treatment protocols, and long-term outcomes highlight the need for standardized, high-quality randomized trials to better define them. In clinical practice, ketamine is primarily used as an anesthetic and analgesic agent. At sub-anesthetic doses, it is also employed for TRD and selected psychiatric or pain conditions rather than as a first-line therapy [22,28,37]. Emerging research on serotonergic psychedelics, including psilocybin and LSD, suggests that these agents may represent complementary therapeutic approaches for depression, although the evidence remains less established than for ketamine [60]. Ketamine’s antidepressant and analgesic effects are observed in both acute and perioperative settings, and it is frequently administered as part of broader treatment strategies [28]. Although ketamine offers important benefits, its administration requires careful patient selection, appropriate monitoring, and consideration of regulatory and safety issues, which are discussed in the following section.

## 5. Regulatory Framework, Safety Concerns, and Future Perspectives

### 5.1. Legal Status and Access

Ketamine’s legal status varies across jurisdictions. As it is not scheduled under the 1971 United Nations Convention on Psychotropic Substances, its regulation is determined at the national level through country-specific scheduling and control frameworks [64]. Although it remains an essential pharmaceutical in anesthesia and emergency care, the drug is widely classified as a controlled substance due to concerns regarding diversion and non-medical use. Accordingly, access outside approved medical or scientific purposes is regulated under national drug control laws governing prescribing, distribution, and administration [22,64].

In the United States, ketamine is classified as a Schedule III controlled substance under the Controlled Substances Act, reflecting its accepted medical usage alongside a recognized potential for misuse and dependence. Its administration is therefore restricted to healthcare settings such as hospitals and licensed clinics [65]. Similarly, in Canada, the compound is classified as a Schedule I controlled substance under the Controlled Drugs and Substances Act, where possession, sale, and production are prohibited unless authorized for medical, scientific, or industrial purposes. Even with stricter scheduling, ketamine retains legitimate clinical applications, mainly in anesthesia [66]. Across Asia and Australia, a similar regulatory trend is observed, where this substance is subject to progressively stricter national control measures in response to increasing concerns about diversion, illicit trafficking, and recreational use [64].

Within Europe, ketamine is not governed by a single harmonized scheduling system but instead regulated through national frameworks. Monitoring data indicate an increasing prominence since the early 2000s, as reflected in rising seizure rates and broader detection in illicit markets. This trend corresponds with a partial shift from its clinical administration as an anesthetic toward mood-elevating use in nightlife venues and clubs [67,68]. Although the prevalence of non-medical exploitation remains low in general population estimates, it is concentrated in specific subpopulations where acute harm and treatment demand have been reported. Alongside these patterns, indicators suggest increasing availability and diversion from legitimate pharmaceutical supply chains. Hence, regulatory responses across European countries focus on maintaining medical access while strengthening national control measures and enforcement practices. However, differences in legal classification and scheduling across Member States continue to create regulatory fragmentation, which may facilitate cross-border diversion and trafficking [68].

Ketamine is not available for over-the-counter purchase. It is administered in controlled healthcare settings under the supervision of qualified professionals, such as anesthesiologists, emergency physicians, and other specialists. Established indications include general anesthesia, procedural sedation, and pain management [37]. As summarized in Table 1, clinical studies have demonstrated its antidepressant efficacy, especially in patients with TRD. Nevertheless, its use in psychiatric indications remains largely off-label and under ongoing investigation [37].

Regardless of the growing evidence supporting the rapid beneficial effects of ketamine in emotional disorders, public awareness of its medical applications remains variable. The drug is still widely associated with recreational use and its dissociative and euphoric sensations, which may contribute to stigma and misconceptions regarding its therapeutic value. Survey-based investigations suggest that regulatory approval of ketamine-derived treatments, such as esketamine for depression, does not necessarily change perceived risk or alter patterns of mood-elevation among individuals who use psychoactive substances [22,28,64]. These findings highlight a gap between advances in scientific knowledge and public understanding of its potential in healthcare.

### 5.2. Safety Considerations

Ketamine is associated with a range of dose-dependent and context-dependent adverse effects. Short-term reactions commonly include dissociation, sedation, transient increases in blood pressure, dizziness, nausea, vomiting, and perceptual disturbances, all of which are typically managed in controlled clinical environments with appropriate monitoring. Neuropsychiatric effects such as anxiety, dysphoria, confusion, hallucinations, and emergence reactions are also well documented, particularly during recovery from anesthesia or higher-dose administration. While the drug generally preserves airway reflexes and maintains spontaneous respiration at therapeutic doses, respiratory depression may occur with rapid administration, high doses, or concomitant use of other central nervous system depressants, necessitating careful monitoring [37,69].

Overall, ketamine is considered well-tolerated under medical supervision. Despite this, repeated or prolonged exploitation has been associated with the development of tolerance and carries a risk of abuse and dependence, especially outside supervised settings. Evidence from studies of chronic recreational ketamine use further indicates that prolonged high-dose exposure may result in significant toxicities and health complications, including ulcerative cystitis and lower urinary tract dysfunction, hepatobiliary and gastrointestinal abnormalities, as well as persistent cognitive and psychiatric disturbances. These adverse outcomes have been reported primarily in individuals using ketamine for entertainment at substantially higher cumulative doses than those employed in clinical environments and therefore should not be directly extrapolated to therapeutic administration. Nevertheless, they point out the importance of careful patient selection and ongoing surveillance during treatment [70]. As the long-term consequences of repeated ketamine administration remain incompletely characterized, further longitudinal studies are needed to clarify its long-term safety.

### 5.3. Future Directions

Future research should prioritize large-scale randomized controlled trials with extended follow-up to clarify the long-term efficacy and safety of ketamine across psychiatric and substance use indications. Particular emphasis should be placed on reducing the substantial heterogeneity in dosing regimens, routes of administration, maintenance protocols, and outcome measures, which currently limit direct comparisons across studies and the development of verified treatment recommendations. Standardized trial designs and rigorous evaluation of ketamine’s role as a fast-acting bridge intervention for acute suicidality will be essential to improve comparability across studies and support clinical translation [26,71]. Further investigation into its neurobiological mechanisms remains essential, with special attention to glutamatergic signaling, synaptic plasticity, neurotrophic pathways, and the potential contribution of adenosine signaling to its antidepressant effects [39,40]. In addition, adequately powered head-to-head trials comparing racemic ketamine with esketamine and established treatments such as ECT and rTMS are needed to better define their relative effectiveness, durability of benefit, and safety. Additional studies should also evaluate the role of KAP as a potential augmentation strategy and identify patient characteristics associated with sustained treatment response [43]. Advancing understanding through rigorous research will be essential to define ketamine’s appropriate clinical role while ensuring patient safety.

## 6. Conclusions

This scoping review highlights ketamine as a clinically significant, rapid-acting psychoactive agent with an important role in modern psychopharmacology. Originally developed as an anesthetic, the drug has gained prominence in psychiatry due to its early antidepressant effects, particularly in TRD and acute suicidal ideation. In contrast to conventional monoaminergic substances, which typically require several weeks to achieve therapeutic benefit, it may induce rapid reductions in depressive symptom severity, with clinically relevant improvements observed in some patients within hours of administration.

The available evidence suggests that ketamine’s pharmacological effects, primarily mediated through NMDAR antagonism and downstream modulation of glutamatergic signaling, support a shift in conceptual models of depression toward synaptic plasticity–based and neurobiological pathway–level mechanisms. Furthermore, while ketamine and its enantiomer esketamine show similar acute efficacy in available comparative studies, differences in the durability of response remain insufficiently defined. Although the existing findings consistently support ketamine’s fast antidepressant properties, interpretation of long-term efficacy remains limited by heterogeneous study designs, variability in therapeutic protocols, and relatively short follow-up periods. Safety considerations, including dissociative and cardiovascular effects, transient cognitive effects, and risks associated with misuse or dependence, necessitate controlled administration and careful patient selection. These factors, combined with regulatory restrictions and off-label applications in psychiatry, highlight the need for cautious clinical implementation.

Future research should prioritize large-scale, long-term randomized controlled trials, standardized treatment protocols, and the identification of predictors of response and sustained benefit. Further evaluation of strategies, including KAP, may help improve the durability of response. Additional work should also clarify optimal dosing strategies, maintenance regimens, and the potential synergistic role of ketamine when combined with psychiatric interventions. Overall, this agent represents a unique therapeutic option and an important translational model for understanding and targeting synaptic and circuit-level mechanisms in mood disorders.

## Figures and Tables

**Table 1:** Clinical efficacy and dosing strategies of ketamine across randomized and observational studies in psychiatric and related disorders ^1^.

Study Design	Population	Intervention	Control	Key Clinical Outcomes	Reference
Randomized, double-blind, placebo-controlled crossover trial	Adults with MDD and antidepressant failure (*n* = 20 randomized; *n* = 18 analyzed)	IN ketamine 50 mg (single dose)	Saline placebo	Greater antidepressant effect than placebo at 24 h (response 44% vs. 6%), but short-term only	[34]
Randomized, double-blind, placebo-controlled trial	Adults with TRD-MDD (*n* = 79 randomized; *n* = 56 analyzed)	IV ketamine 0.1, 0.5, or 1.0 mg/kg (single infusion)	Active comparator (midazolam)	Rapid reduction in suicidal ideation; effects diminished over time, with lower scores than midazolam at 30 days.	[45]
Randomized, double-blind, active-controlled trial	Adults with TRD (*n* = 58 randomized; *n* = 54 analyzed)	IV ketamine 0.5 mg/kg (6 infusions over 12 days)	Active comparator (midazolam)	Greater MADRS reduction during mid-treatment vs. comparator, no difference at endpoint	[46]
Randomized, double-blind, active-controlled trial	Adults with TRD (*n* = 33 randomized; *n* = 20 analyzed)	IV ketamine 0.5 mg/kg (daily for 3 days)	Active comparator (midazolam)	Both ketamine and midazolam groups showed significant improvement within-group MADRS, but no significant between-group difference	[47]
Single-arm, open-label phase II trial	Adults with MDD and advanced cancer in palliative care (*n* = 20 analyzed)	IN ketamine 50–150 mg (3 doses over 1 week)	None	Marked short-term symptom reduction (response 70%; remission 45%), but uncontrolled design limits interpretation	[48]
Randomized, double-blind, placebo-controlled dose-ranging trial	Adults with TRD (*n* = 67 randomized; *n* = 67 analyzed)	IN esketamine 28 mg, 56 mg or 84 mg (twice weekly with maintenance)	Placebo + oral antidepressant	Dose-dependent reduction in depressive symptoms vs. placebo with sustained benefit up to ~9 weeks	[21]
Systematic review and meta-analysis of randomized control trials	Adults with unipolar or bipolar depression (20 trials)	Single and repeated ketamine administration	Placebo or active control	Significant antidepressant effect peaking at 24 h, sustained up to ~7 days, enhanced with repeated dosing	[49]
Systematic review (dosing optimization across clinical use)	Mixed populations (anesthesia, pain, psychiatric disorders)	IV/IM/IN/oral/SC ketamine; variable dosing strategies	Comparative dosing regimens	Evidence supports 0.5 mg/kg IV (depression) and 0.1–0.3 mg/kg slow IV (analgesia); slow infusion + individualized dosing improves safety; rapid/high doses increase adverse effects	[26]

^1^ MDD: major depressive disorder; TRD: treatment-resistant depression; MADRS: Montgomery–Åsberg Depression Rating Scale; IV: intravenous; IM: intramuscular; IN: intranasal; SC: subcutaneous.

**Table 2:** Comparative clinical characteristics of treatments used across major depressive disorder (MDD) and treatment-resistant depression (TRD) [27,50,51,52,53,54,55,56] ^1^.

Feature	Ketamine (Intravenous)	Esketamine (Intranasal)	SSRIs/SNRIs	ECT	rTMS
**Main evidence population**	Primarily investigated in TRD; also studied in severe MDD	Primarily investigated and approved for TRD	Broad MDD population; commonly used before TRD designation	Severe depression, including TRD/refractory depression and episodes with acute suicidal ideation	Mainly TRD and selected MDD populations
**Onset of antidepressant effect**	Very rapid (hours–days)	Very rapid (hours–days)	Usually delayed (weeks to months)	Rapid (often 1–2 weeks during an acute course)	Usually gradual (weeks)
**Evidence in TRD**	Consistent evidence of short-term efficacy; comparative superiority uncertain	Demonstrated efficacy in TRD; approved for this indication in several jurisdictions	Reduced efficacy after multiple unsuccessful antidepressant trials	Established effective treatment for refractory/TRD depression; particularly effective in severe episodes	Moderate-to-high efficacy in selected TRD populations
**Effect on suicidal ideation**	Rapid reductions in suicidal symptoms reported; persistence of effects requires further study	Rapid reductions in suicidal symptoms reported in acute settings; long-term effects require maintenance strategies	Reduction in suicidal ideation generally occurs through improvement of depressive symptoms over time	Reduction in suicidal symptoms demonstrated in severe depression; effects require consideration of continuation strategies	Reduction in suicidal ideation demonstrated in clinical trials and meta-analysis
**Duration of benefit**	Benefits may diminish without repeated dosing or maintenance strategies	Continued administration is generally required to maintain response after induction	Continued treatment reduces relapse risk among responders	Relapse after acute ECT is common; continuation/maintenance ECT may prolong remission in selected patients	Durability varies according to stimulation protocol, patient characteristics, and maintenance schedules
**Primary mechanism**	NMDA receptor antagonism with downstream glutamatergic modulation and neuroplastic effects	S-enantiomer NMDAR antagonism with downstream glutamatergic and neuroplastic effects	Target monoaminergic systems: serotonin (SSRIs) and serotonin–norepinephrine (SNRIs)	Anesthesia-assisted seizure induction causing therapeutic neurobiological changes	Modulation of neuronal cortical networks through magnetic stimulation

^1^ NMDAR: N-methyl-D-aspartate receptor; SSRIs: selective serotonin reuptake inhibitors; SNRIs: serotonin–norepinephrine reuptake inhibitors; ECT: electroconvulsive therapy; rTMS: repetitive transcranial magnetic stimulation.

## Data Availability

No new data were created or analyzed in this study. Data sharing is not applicable to this article.

## Supplementary Materials

The following supporting information can be downloaded at: https://www.mdpi.com/article/10.3390/jcm15155922/s1, File S1: Preferred Reporting Items for Systematic reviews and Meta-Analyses extension for Scoping Reviews (PRISMA-ScR) Checklist; Table S1: Complete electronic search strategies for all databases and additional information sources used to identify eligible studies. Database-specific controlled vocabulary (e.g., MeSH terms in PubMed) and free-text terms were combined using Boolean operators, as appropriate for each database.

## Abbreviations

The following abbreviations are used in this manuscript:

AMPA	*α*-Amino-3-Hydroxy-5-Methyl-4-Isoxazolepropionic Acid
BDNF	Brain-Derived Neurotrophic Factor
ECT	Electroconvulsive Therapy
eEF2	Eukaryotic Elongation Factor 2
FDA	United States Food and Drug Administration
GABA	*γ*-Aminobutyric Acid
KAP	Ketamine-Assisted Psychotherapy
LSD	Lysergic Acid Diethylamide
MDD	Major Depressive Disorder
mTOR	Mechanistic Target of Rapamycin
mTORC1	Mechanistic Target of Rapamycin Complex 1
NMDAR	N-Methyl-D-Aspartate Receptor
PRISMA-ScR	Preferred Reporting Items for Systematic Reviews and Meta-Analyses Extension for Scoping Reviews
rTMS	Repetitive Transcranial Magnetic Stimulation
SNRIs	Serotonin–Norepinephrine Reuptake Inhibitors
SSRIs	Selective Serotonin Reuptake Inhibitors
TRD	Treatment-Resistant Depression
TrkB	Tropomyosin Receptor Kinase B
