# Supplementary material for: Psychoactive and Neurobiological Effects of Ketamine in Humans: A Scoping Review of Clinical Evidence
Source: J Clin Med. 2026 Jul 29;15(15):5922. doi: 10.3390/jcm15155922 (PMC13467468; doi:10.3390/jcm15155922)
Supplement: Supplementary file 1 [file jcm-15-05922-s001.zip › Table S1.pdf]

Supplementary Material

Psychoactive and Neurobiological Effects of Ketamine in Humans: A Scoping Review of Clinical Evidence

Sidorela Turtulli [1] and Eugenia Papadaki [1, 2, *]

[1] Department of Biomedical Science, School of Health and Sports Sciences, New York College, 138 Egnatia, 54622 Thessaloniki, Greece; sturtulli@nyc.gr

[2] Department of Human Nutrition and Dietetics, School of Health and Sports Sciences, New York College, 138 Egnatia, 54622 Thessaloniki, Greece

* Correspondence: epapadaki@nyc.gr; Tel: +30-2310889879

Supplementary Table S1. Complete electronic search strategies for all databases and additional information sources used to identify eligible studies. Database-specific controlled vocabulary (e.g., MeSH terms in PubMed) and free-text terms were combined using Boolean operators, as appropriate for each database.

Database	Search strategy
**PubMed**	Search fields: MeSH terms and Title/Abstract (tiab)<br>Search string:<br>(("Ketamine"[Mesh] OR ketamine[tiab] OR esketamine[tiab] OR "S-ketamine"[tiab])<br>AND<br>("Depressive Disorder"[Mesh] OR depression[tiab] OR "major depressive disorder"[tiab] OR "treatment-resistant depression"[tiab] OR TRD[tiab] OR antidepress*[tiab] OR psychiat*[tiab] OR psychoactive[tiab] OR pharmacology[tiab] OR neuropharmacology[tiab] OR neurobiology[tiab] OR mechanism*[tiab] OR pharmacokinetic*[tiab] OR metabolism[tiab] OR glutamate[tiab] OR glutamatergic[tiab] OR NMDA[tiab] OR NMDAR[tiab] OR AMPA[tiab] OR BDNF[tiab] OR TrkB[tiab] OR mTOR[tiab] OR ERK[tiab] OR eEF2[tiab] OR adenosine[tiab] OR anesthesia[tiab] OR analgesia[tiab] OR safety[tiab] OR toxicity[tiab] OR "clinical trial"[Publication Type] OR trial*[tiab] OR "systematic review"[Publication Type]))<br>Filters: English; Humans<br>Search period: Database inception to 19 July 2026<br>Date last searched: 19 July 2026
**Scopus**	Search field: TITLE-ABS-KEY<br>Search string:<br>TITLE-ABS-KEY((ketamine OR esketamine OR "S-ketamine")<br>AND<br>(depression OR "major depressive disorder" OR "treatment-resistant depression" OR TRD OR antidepress* OR psychiatr* OR psychoactive OR pharmacology OR neuropharmacology OR neurobiology OR mechanism* OR pharmacokinetic* OR metabolism OR glutamate OR glutamatergic OR NMDA OR NMDAR OR AMPA OR BDNF OR TrkB OR mTOR OR ERK OR eEF2 OR adenosine OR anesthesia OR analgesia OR safety OR toxicity OR "clinical trial" OR trial* OR "systematic review"))

	Filters: English; Article; Review<br>Search period: Database inception to 19 July 2026<br>Date last searched: 19 July 2026
**Web of Science**	Search field: Topic (TS)<br>Search string:<br>TS=((ketamine OR esketamine OR "S-ketamine")<br>AND<br>(depression OR "major depressive disorder" OR "treatment-resistant depression" OR psychiat* OR psychoactive OR neurobiology OR neuropharmacology OR pharmacology OR glutamate OR glutamatergic OR NMDA OR NMDAR OR anesthesia OR analgesia))<br>Indexes searched: Science Citation Index Expanded (SCI-EXPANDED), Social Sciences Citation Index (SSCI), and Emerging Sources Citation Index (ESCI)<br>Filters: English<br>Search period: Database inception to 19 July 2026<br>Date last searched: 19 July 2026
**Google Scholar**	Searches performed individually:<br>ketamine depression<br>ketamine treatment-resistant depression<br>ketamine pharmacology<br>ketamine neuropharmacology<br>ketamine mechanism of action<br>ketamine psychiatric disorders<br>ketamine anesthesia depression<br>ketamine psychoactive effects humans<br>ketamine clinical trial<br>esketamine depression<br>ketamine glutamatergic signaling<br>Results were screened in order of relevance until no additional eligible studies meeting the pre-defined inclusion criteria were identified.<br>Filters: English<br>Search period: Database inception to 19 July 2026<br>Date last searched: 19 July 2026
**Additional searches**	Reference lists of included studies and relevant reviews were screened manually. Backward citation tracking was performed to identify additional eligible sources.<br>Backward citation tracking was performed.<br>Official websites of the World Health Organization (WHO), European Medicines Agency (EMA), European Union Drugs Agency (EUDA), United States Drug Enforcement Administration (DEA), and Health Canada were consulted to identify relevant regulatory and public health information.<br>Date last consulted: 19 July 2026
